# A retrospective study of Reyanning mixture in elderly patients infected with SARS-CoV-2 Omicron variant

**DOI:** 10.3389/fphar.2023.1185122

**Published:** 2023-07-20

**Authors:** Changya Liu, Xinxin Wu, Hongqiang Yang, Xiangru Xu, Caiyu Chen, Linguangjin Wu, Wen Zhang, Haimei Shi, Yuerong Fei, Yuting Sun, Hongze Wu, Shuang Zhou, Bangjiang Fang

**Affiliations:** ^1^ Department of Emergency, LongHua Hospital, Shanghai University of Traditional Chinese Medicine, Shanghai, China; ^2^ Shanghai Skin Disease Hospital, Tongji University Skin Disease Hospital, Shanghai, China; ^3^ Jiangxi Provincial Traditional Chinese Medicine Nephropathy Clinical Research Center, Jiujiang Hospital of Traditional Chinese Medicine, Jiujiang, Jiangxi, China; ^4^ Acupuncture and Massage College, Shanghai University of Traditional Chinese Medicine, Shanghai, China; ^5^ Institute of Emergency and Critical Care Medicine, Shanghai University of Traditional Chinese Medicine, Shanghai, China

**Keywords:** Reyanning mixture, elderly patients, hospitalization duration, COVID-19, Omicron variant, viral shedding time

## Abstract

**Objective:** Reyanning mixture has been demonstrated to be effective in treating infected patients during the outbreak pandemic of SARS-CoV-2 Omicron variant of Coronavirus disease 2019 (COVID-19) in Shanghai 2022. The aim of this study is to further investigate the role of Reyanning mixture specifically in the treatment of elderly patients.

**Methods:** This study enrolled 1,102 elderly patients who were infected with SARS-CoV-2 Omicron variant. Of these, 291 patients received Reyanning mixture in conjunction with conventional Western medicine treatment were assigned to the treatment group, while 811 patients only received conventional Western medicine treatment were assigned to the control group. Clinical parameters including hospitalization duration, viral shedding time, and Cycle Threshold (Ct) values of novel coronavirus nucleic acid tests, as well as adverse events were recorded and analyzed in both groups.

**Results:** There was no significant difference in baseline characteristics between two groups. In comparison to the control group, the treatment group demonstrated a substantial difference in hospitalization duration (median: 8 days vs. 10 days, HR: 0.638, 95% CI: 0.558–0.731, *p* < 0.001). The treatment group also showed a significantly shorter viral shedding time compared to the control group (median: 7 days vs. 8 days, HR: 0.754, 95% CI: 0.659–0.863, *p* < 0.001). Multivariate Cox proportional-hazards model analysis indicated that the use of Reyanning mixture was closely associated with a reduction in hospitalization duration (HR: 1.562, 95% CI: 1.364–1.789, *p* < 0.001) and viral shedding time (HR: 1.335, 95% CI: 1.166–1.528, *p* < 0.001). In addition, during the treatment process, no serious adverse event occurred in either group.

**Conclusion:** The improvement of clinical parameters in the treatment group indicate a promising therapeutic benefit of Reyanning mixture for elderly patients infected with SARS-CoV-2 Omicron variant in the present study. Further investigations are required to validate this finding by examining the underlying mechanism and function of Reyanning mixture.

## Introduction

The outbreak pandemic of COVID-19 has raised significant impact to public health, resulted in widespread suffering across the globe (Teng et al., 2021; Zhang X. et al., 2022; Huang et al., 2022). During this period, vaccines and medications have been developed and implemented to combat the viral infection (Wang Y. et al., 2020; Xu et al., 2022b; Ciotti et al., 2022; Wong et al., 2022), and the SARS-CoV-2 Omicron variant infection appeared to become less virulent. However, the health of elderly people continues to be challenged, as they are more susceptible to severe outcome subsequent to infection (Zhang et al., 2022a), and available data indicated that advanced age and underlying health conditions confer the greatest risk for developing severe COVID-19 infection, which is linked to increased morbidity and mortality rates (Huemer et al., 2021; Ramasamy et al., 2021; Chen et al., 2022; Tong et al., 2022).

Traditional Chinese medicine (TCM) has been widely recognized as medical science with thousands of years, and incorporated as one of the therapeutic options for addressing COVID-19. Despite studies demonstrating the promising therapeutic effects of TCM and integrated traditional Chinese-Western medicine to treat COVID-19 (Yang Y. et al., 2020; Luo et al., 2020; Zhong et al., 2022), there is limited research specifically focus on the elderly patients’ treatment, which is closely associated with reducing the incidence of severe disease and mortality. Reyanning mixture is a Chinese patent medicine, which composed of four botanical drugs: *Taraxacum mongolicum* Hand.-Mazz. [Asteraceae, Herba taraxaci], *Reynoutria japonica* Houtt. [Polygonaceae, Polygoni cuspidati rhizoma et radix], *Sonchus brachyotus* DC. [Asteraceae, Sonchus arenicola Vorosch.], and *Scutellaria barbata* D.Don [Lamiaceae, Herba scutellariae barbatae], has been recommended in “The Diagnosis and Treatment of New Coronavirus Infected Pneumonia of Shaanxi Province (Trial edition 2)” (Li et al., 2021b). And the efficacy in treating SARS-CoV-2 infection has been illustrated in our previous research (Xu et al., 2023). The objective of the current investigation is to examine the correlation between administration of Reyanning mixture and clinical outcomes among elderly patients.

## Methods and materials

### Recruitment of participants

All data were obtained from individuals who were hospitalized during the pandemic outbreak in Shanghai 2022. The dataset comprised demographic data, medical history, illness status, hospitalization duration, and results from coronavirus nucleic acid testing. This study was applied in compliance with the tenets of Good Clinical Practice and the Declaration of Helsinki, granted ethical approval by the Medical Ethics Committee (Approval number: JJSZYYY20220403) and was based on the registered clinical trial (ChiCTR2200060292). Informed consent has been collected from all patients.

The diagnostic criteria were applied according to the ninth trial edition guidelines for the diagnosis and treatment of COVID-19 (General Office of National Health Commission of the People’s Republic of China, Office of National Administration of Traditional Chinese Medicine, 2022), as outlined below:

Diagnostic criteria.

1. Presence of relevant epidemiological history.
2. Presence of at least two of the following mentioned clinical symptoms:
  (i) fever and/or respiratory symptoms, or other clinical manifestations of COVID-19;
  (ii) imaging features in accordance with COVID-19;
  (iii) A normal or decreased total white blood cell count as well as lymphocyte count in the early stage of illness.
3. Presented with one of the following microbiological or serological evidence:
  (i) novel coronavirus nucleic acid detected positively;
  (ii) positive detection of both IgM and IgG antibodies to novel coronavirus in patients who have not been vaccinated.

Clinical presentations were also assessed according to the guideline criteria (General Office of National Health Commission of the People’s Republic of China, Office of National Administration of Traditional Chinese Medicine, 2022), and an classification of the severity of the illness is presented below.

1. Mild: A mild clinical symptom without image evidence of pneumonia.
2. Common: Clinical manifestations mentioned above, in addition to image evidence of pneumonia.
3. Severe: Individuals who met one or more criteria as follow:
  (i) Shortness of breath, respiratory rate ≥30 breaths/minute;
  (ii) Oxygen saturation equal or below to 93% in a resting state;
  (iii) Arterial partial pressure of oxygen (PaO2)/fraction of inspired oxygen (FiO2) ≤300 mmHg;
  (iv) Progressive worsening of clinical symptoms and pulmonary imaging showing significant lesion progression >50% within 24–48 h.
4. Critical: Met any condition as follow:
  (i) Individuals who suffered respiratory failure with requirement of mechanical ventilation;
  (ii) Shock;
  (iii) Individuals who suffered multiple organ dysfunction or failure and required intensive care.

We screened the data of all COVID-19 patients who received treatment during hospitalization and finally obtained the cases for this study, based on the criteria as follow:

Inclusion criteria.

1. An individual with an age greater than or equal to 60 years;
2. Patients diagnosed with mild or asymptomatic type of COVID-19 according to the diagnostic criteria;
3. Only received conventional Western medical therapy or combine with Reyanning mixture.

Exclusion criteria.

1. Diagnosed with severe type of COVID-19;
2. Deterioration of clinical symptoms or death within 48 h of admission;
3. Those who were suffering from severe underlying diseases;
4. A severe psychiatric disorder and medication was required;
5. Patient who received other TCM botanical drugs or participated in any other clinical trial beside Reyanning mixture;
6. Received any kind of antiviral, corticosteroid, or monoclonal antibody;
7. Discontinuation, intolerance or refusal to take Reyanning mixture.

The screened and enrolled patients were split into two groups. The treatment group was given Reyanning mixture combine with conventional Western medicine therapy, while the control group received conventional Western medicine based on the Guideline (General Office of National Health Commission of the People’s Republic of China, Office of National Administration of Traditional Chinese Medicine, 2022). Furthermore, all participants were categorized into three subgroups according to their age.

Stratum Ⅰ: Age equal to/greater than 60 years and less than 65 years (≥60 years and <65 years).

Stratum Ⅱ: Age equal to/greater than 65 years and less than 70 years (≥65 years and <70 years).

Stratum Ⅲ: Age equal to/greater than 70 years (≥70 years).

### Investigational medications

As a Type A extract (Heinrich et al., 2022), Reyanning mixture was produced by Xingfu Pharmaceutical Group Co., Ltd. (Xi’an, Shaanxi, China), received approval from the National Medical Product Administration of China in 2005 (Approval number: Z20050493), and has been included in the Chinese Pharmacopoeia. According to the Chinese Pharmacopoeia (2020 edition), pharmaceutical manufacturer obtained 372 g *Taraxacum mongolicum* Hand.-Mazz. (Asteraceae, Herba taraxaci), 372 g *Reynoutria japonica* Houtt. [Polygonaceae, Polygoni cuspidati rhizoma et radix], 372 g *Sonchus brachyotus* DC. [Asteraceae, Sonchus arenicola Vorosch.], and 186 g *Scutellaria barbata* D.Don [Lamiaceae, Herba scutellariae barbatae]. The four botanical drugs mentioned above were decocted twice with water. The first decoction lasted for 2 hrs, and the second for 1 h. The decoction was filtered, concentrated under reduced pressure to an appropriate volume and combined, centrifuged, filtered, and heated to boiling. Finally, 1,000 mL of the decoction was obtained. The quality control analysis of Reyanning mixture used high performance liquid chromatography (HPLC) (Heinrich et al., 2022) has been reported by Su et al. (2021). The primary metabolites of the Reyanning mixture including polydatin, emodin, luteolin, caffeic acid, and chlorogenic acid, have been qualitatively controlled and quantitatively assessed (Su et al., 2021), which were consistent with the Medicine Standards stipulated by the National Medical Products Administration of China.

The active metabolites such as chlorogenic acid, emodin, and caffeic acid, have been studied for their potential antiviral activity against COVID-19 (Adem et al., 2021; Shao et al., 2022; Wang et al., 2022). Polydatin has been found to bind Spike, ACE2, and ACE2, thereby hindering SARS-CoV-2 (Perrella et al., 2021). Moreover, luteolin has been experimentally evaluated against SARS-CoV-2’s RNA-dependent RNA polymerase (Munafò et al., 2022), demonstrating its potential therapeutic effect for COVID-19.

The administration of Reyanning mixture involved oral dosages of 20 mL four times daily, started from the day of enrollment and continued for seven consecutive days. The remaining hospitalization period was followed by standard treatment. A detailed record was kept of the concurrent medications. The hospitalized patients were monitored until discharge, while patients who were discharged within 7 days were subjected to telephone follow-ups to monitor and report any unfavorable incidents. Patients who met the discharge criteria or experienced deterioration of the condition and required hospital transfer were considered to have fulfilled the criteria for study completion.

### Evaluation of clinical outcomes

In both groups, pharyngeal swab NATs were performed daily for all the patients. The criteria for discharge was defined according to the Guideline (General Office of National Health Commission of the People’s Republic of China, Office of National Administration of Traditional Chinese Medicine, 2022) as follows: 1) Body temperature maintained normal for at least three consecutive days; 2) Respiratory symptoms improved significantly; 3) Significant improvement in acute infiltrative lesions manifested with pulmonary imaging; 4) Two consecutive COVID-19 nucleic acid tests of N gene and ORF gene with a Ct value of ≥35, or two consecutive negative nucleic acid tests (a minimum sampling interval of 24 h was required).

The primary outcome for this study was hospitalization duration and viral shedding time. The hospitalization duration was measured from admission to the date of discharge. Viral shedding time was measured from the first positive result to the date on which the second consecutive negative nucleic acid test result was obtained. Additionally, the Ct values of the ORF and N genes between two groups were analyzed. The secondary outcome was the evaluation of the overall adverse events, adverse events related to the use of Reyanning mixture, as well as the deterioration of illness during the treatment process. All adverse events that occurred during the treatment process were closely monitored by physicians, while the severity, duration, and onset time were carefully recorded.

### Statistical analysis

The analyses of the continuous variables (presented as medians, interquartile ranges) were conducted with the Mann-Whitney U test, while the analyses of categorical variables were conducted with the Chi-square test for counts and percentages (%). Cox proportional-hazards models and 95% confidence intervals (CI) were applied to estimate the variables that may impact the outcome. The Kaplan-Meier method was applied to present the time to events with a 95% CI and generate survival curves.

We used Cox proportional-hazards models to perform sensitivity analysis. Firstly, model 1 was established by adjusting only for age to explore the impact of Reyanning mixture on outcome. Then, model 2 was established by adjusting for patient characteristics listed in our study. Next, we selected cases with restricted age range based on age stratification and re-analyzed the association between Reyanning mixture and outcome. Statistical significance was determined by a *p*-value less than 0.05 in all tests. Statistical analyses were applied with SPSS (version 26.0; IBM Corp.) and R software (version 4.2.1; R Foundation for Statistical Computing).

## Results

### Baseline and clinical characteristics

**FIGURE 1:**
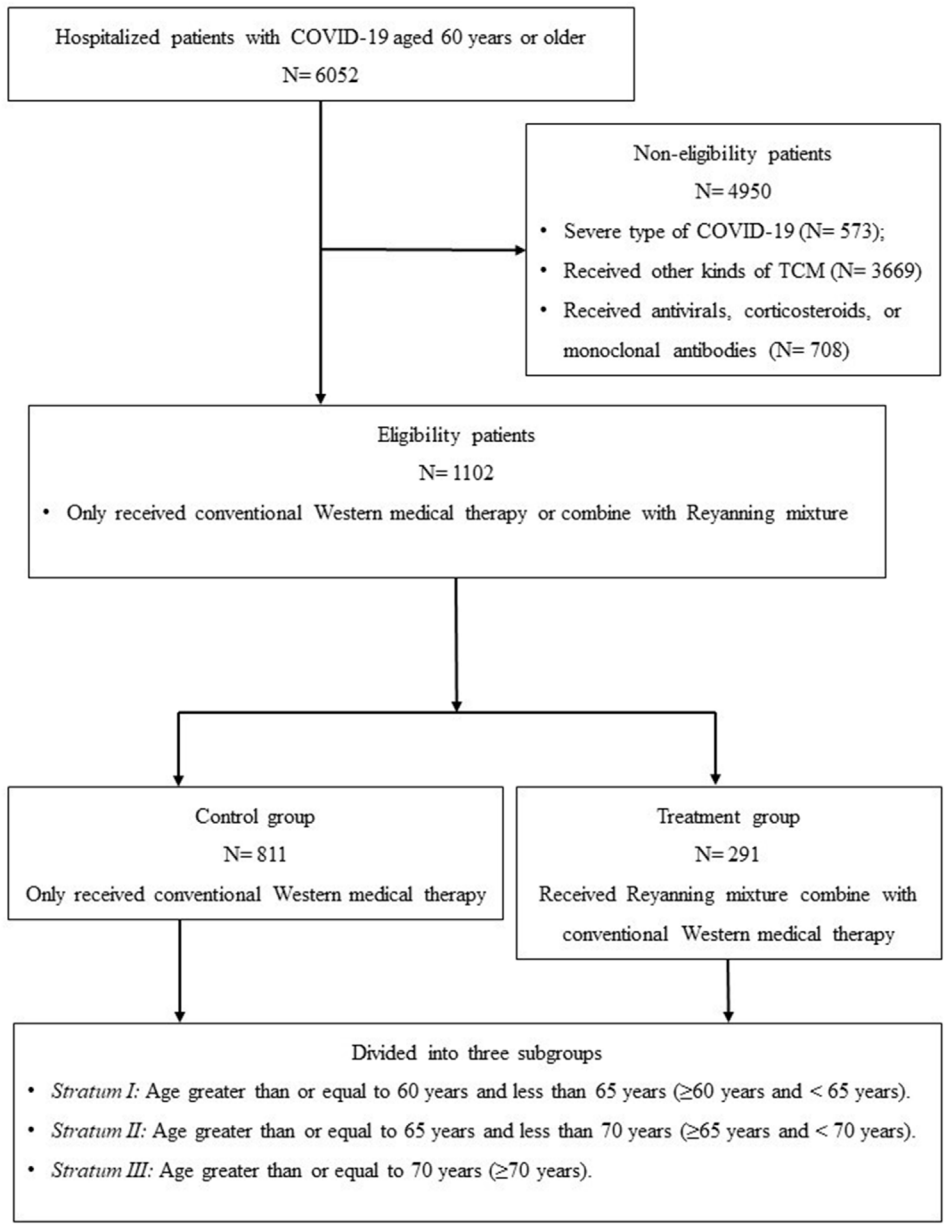
The patient screening flow chart.

Between April 1 and 31 May 2022, a total of 6,052 patients aged 60 years or older were diagnosed with SARS-CoV-2 Omicron infection and admitted to the N3 Mobile Cabin Hospital at the Shanghai New International Expo Center. Among these patients, 1,102 met the inclusion criteria and were collected in this study. Of these patients, 291 received Reyanning mixture combined with conventional Western medical therapy were assigned to the treatment group, while the remaining 811 patients received only conventional Western medical therapy and served as the control group. Flow chart for screening was present in Figure 1.

In the treatment group, the cohort was comprised of 150 males and 141 females, with a median age of 65 (62, 68) years. The control group consisted of 478 males and 333 females, with a median age of 64 (63, 67) years. No statistically different was observed between two groups. A total of 109 patients (37.5%) in the treatment group and 255 (31.4%) in the control group had underlying medical conditions, including hypertension, diabetes, cerebrovascular diseases, and cardiovascular diseases, et al., and there was no statistically significant difference between the two groups (*p* = 0.061). Patients in both groups presented with various clinical syndromes. The most common symptoms of hospitalization included cough, sputum, sore throat, fever, muscle soreness, and fatigue. Regarding vaccination status, 92 patients were unvaccinated, 7 patients were partially vaccinated, 75 received fully dose and 117 received booster within treatment group. In the control group, 235 patients were unvaccinated, 14 had received one dose, 235 had received two doses, and 327 had received a booster. The baseline information of two groups were presented in Table 1.

**TABLE 1:** Baseline information of Treatment group and Control group.

	Treatment group (*n* = 291)	Control group (*n* = 811)	*p-value*
Gender n (%)			
Female	141 (48.5%)	333 (41.1%)	0.985
Age, median (interquartile ranges)	65 (62, 68)	64 (63, 67)	0.453
Underlying diseases n (%)	109 (37.5%)	255 (31.4%)	0.061
Vaccination status n (%)			0.614
Unvaccinated	92 (31.6%)	235 (28.9%)	
Partially vaccinated	7 (2.4%)	14 (1.7%)	
Full vaccination	75 (25.8%)	235 (28.9%)	
Booster	117 (40.2%)	327 (40.3%)	
Type			0.108
Mild	228 (78.4%)	670 (82.6%)	
Asymptomatic	63 (21.6%)	141 (17.4%)	
Clinical symptoms n (%)			
Cough	182 (62.5%)	533 (65.7%)	0.330
Sputum	51 (17.6%)	161 (19.9%)	0.388
Sore throat	52 (17.9%)	108 (13.3%)	0.059
Fever	97 (33.3%)	226 (27.9%)	0.079
Muscle soreness	63 (21.6%)	161 (19.9%)	0.513
Fatigue	42 (14.4%)	103 (12.7%)	0.453
Shortness of breath	7 (2.4%)	18 (2.2%)	0.855
Apocleisis	8 (2.7%)	20 (2.5%)	0.792

### Primary outcome

**FIGURE 2:**
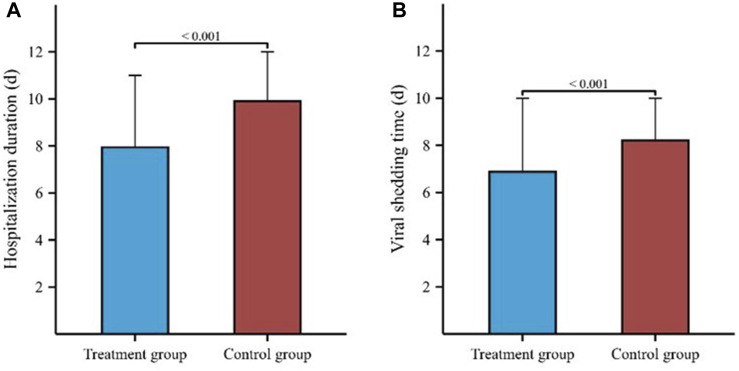
Comparison of hospitalization duration and viral shedding time between Treatment group and Control group. **(A)** represented the comparison of hospitalization duration between two groups. **(B)** represented the comparison of viral shedding time between two groups.

**FIGURE 3:**
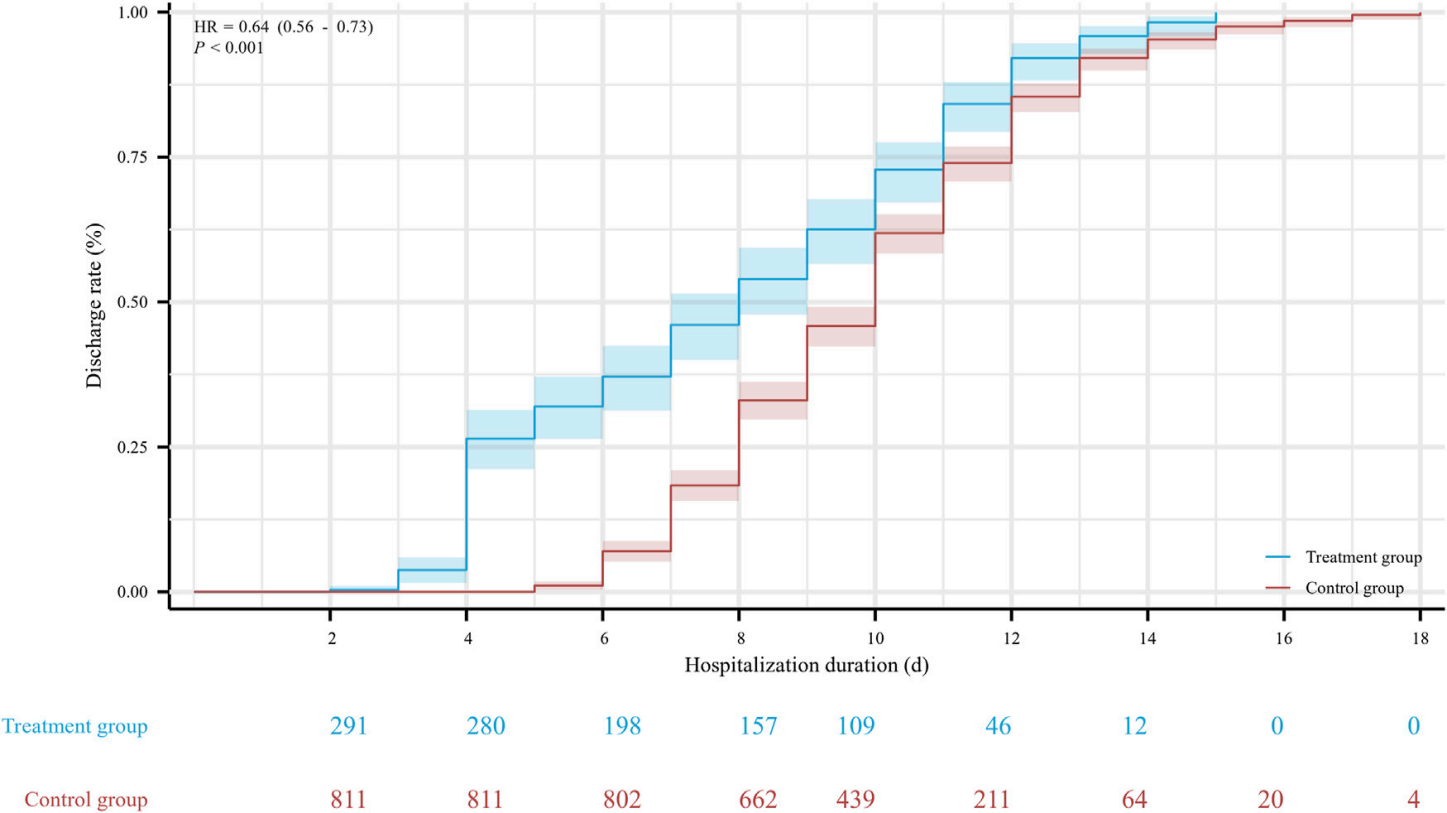
The Kaplan-Meier gram for the hospitalization duration between Treatment group and Control group. HR, hazard ratio.

**FIGURE 4:**
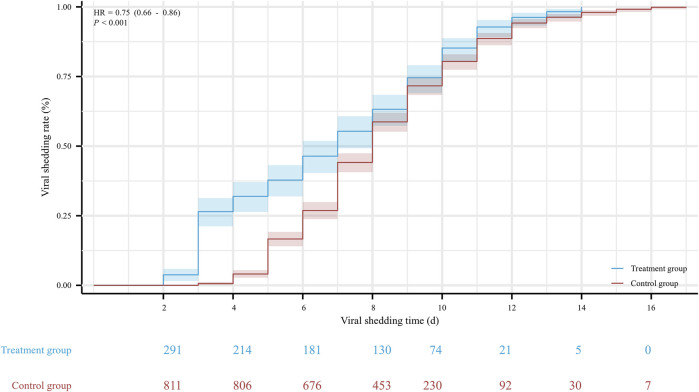
The Kaplan-Meier gram for the viral shedding time between Treatment group and Control group. HR, hazard ratio.

**FIGURE 5:**
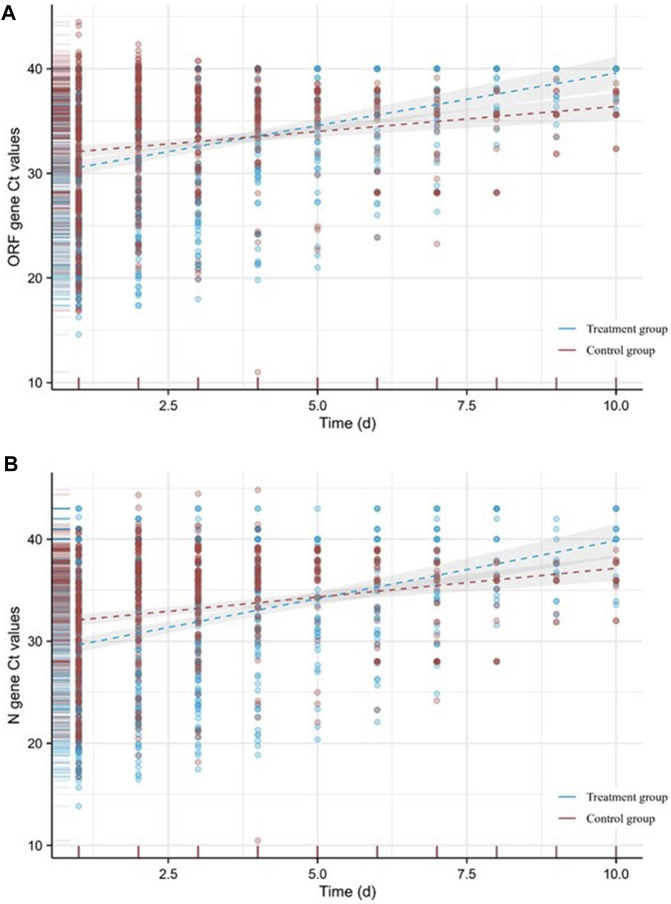
Variation of Ct values of the ORF **(A)** and N **(B)** genes.

The hospitalization duration in the treatment group and the control group were 8 (4, 11) days and 10 (8, 12) days, respectively. Furthermore, the viral shedding time was 7 (3, 10) days in the treatment group and 8 (6, 10) days in the control group, as illustrated in Figure 2. The Kaplan-Meier graph indicated a significant reduction in both hospitalization time and virus clearance time for the treatment group compared to the control group (HR: 0.638, 95% CI: 0.558-0.731, *p* < 0.001; HR: 0.754, 95% CI: 0.659-0.863, *p* < 0.001, respectively) (Figures 3, 4). Furthermore, we collected and analyzed the nucleic acid test results of patients during their hospitalization for a period of 10 days. Both groups of patients exhibited an upward trend in Ct values of the open reading frame (ORF) and nucleocapsid (N) genes after receiving treatment. Comparison with the control group, the results revealed that patients who received treatment with Reyanning mixture exhibited a more pronounced upward trend in Ct values (Figure 5; Table 2).

**TABLE 2:** Comparison of the ORF/N genes Ct values between the Treatment group and Control group.

Day	ORF gene		*p-value*	N gene		*p-value*
	Treatment group	Control group		Treatment group	Control group	
1	30.56 ± 7.48	30.28 ± 5.99	0.579	30.07 ± 7.87	29.73 ± 5.69	0.515
2	31.91 ± 6.99	32.71 ± 5.35	0.094	31.40 ± 7.46	32.36 ± 5.57	0.057
3	32.51 ± 6.62	33.35 ± 4.64	0.066	32.04 ± 7.11	33.29 ± 4.86	0.001
4	32.36 ± 5.97	33.85 ± 4.39	0.001	31.79 ± 6.29	33.56 ± 4.57	0.001
5	33.24 ± 5.62	34.41 ± 4.18	0.001	32.63 ± 5.89	34.48 ± 4.49	0.001
6	34.87 ± 5.17	34.89 ± 3.82	0.955	34.48 ± 5.59	34.75 ± 3.94	0.593
7	35.89 ± 4.45	34.84 ± 3.58	0.020	35.42 ± 4.88	34.61 ± 3.74	0.112
8	37.35 ± 3.28	35.10 ± 3.58	0.001	37.06 ± 3.91	34.77 ± 3.63	0.001
9	38.50 ± 2.36	36.02 ± 2.95	0.001	38.30 ± 2.97	35.80 ± 3.19	0.001
10	38.92 ± 1.73	36.14 ± 2.49	0.001	38.79 ± 2.64	36.03 ± 2.63	0.001

The variables of all enrolled patients that may impact hospitalization duration and viral shedding time, including age, gender, disease type, underlying condition, vaccination status, and use of Reyanning mixture, were incorporated into a Cox proportional-hazards model (presented in Table 3), which revealed a significant association between age, vaccination status, the use of Reyanning mixture with hospitalization duration, as well as viral shedding time (*p* < 0.05). After adjustment for age and vaccination status in the multivariate regression analysis, the result suggested that use of Reyanning mixture significantly benefitted the reduction of both hospitalization duration and viral shedding time (HR: 1.562, 95% CI: 1.364-1.789, *p* < 0.001; HR: 1.335, 95% CI: 1.166-1.528, *p* < 0.001, respectively).

**TABLE 3:** Cox proportional-hazards model for hospitalization duration and viral shedding time.

	Hospitalization duration				Viral shedding time			
	Univariate		Multivariate		Univariate		Multivariate	
	HR (95% CI)	p-value	HR (95% CI)	p-value	HR (95% CI)	p-value	HR (95% CI)	p-value
Age	0.962 (0.945, 0.980)	<0.001	0.963 (0.946, 0.981)	<0.001	0.962 (0.945, 0.980)	<0.001	0.964 (0.947, 0.982)	<0.001
Gender	1.026 (0.911, 1.154)	0.676	0.997 (0.884, 1.126)	0.967	1.045 (0.928, 1.177)	0.465	1.010 (0.895, 1.140)	0.867
Type	1.116 (0.958, 1.299)	0.159	1.146 (0.980, 1.339)	0.088	1.104 (0.948, 1.285)	0.204	1.121 (0.959, 1.310)	0.152
Underlying diseases	1.008 (0.889, 1.143)	0.902	0.948 (0.833, 1.079)	0.417	1.019 (0.899, 1.155)	0.770	0.965 (0.848, 1.098)	0.589
Vaccination status	1.205 (1.059, 1.373)	0.005	1.205 (1.057, 1.373)	0.005	1.189 (1.045, 1.354)	0.009	1.180 (1.035, 1.344)	0.013
Group	1.520 (1.329, 1.740)	<0.001	1.562 (1.364, 1.789)	<0.001	1.307 (1.142, 1.495)	<0.001	1.335 (1.166, 1.528)	<0.001

CI, confidence interval; HR, hazard ratio.

In the sensitivity analysis, firstly, we used hospitalization duration as the dependent variable, while group and age as independent variables, and fitted in the Cox proportional-hazards model 1. The result showed a statistical significance, with a β value of 0.433. Then, we included group, age, gender, vaccination status, underlying diseases, and disease type as independent variables and fitted in the Cox proportional-hazards model 2. The result showed a β value of 0.446, with *p* < 0.001, which was consistent with model 1. Similarly, we applied the above method to analyze the association between group and viral shedding time, and the results showed a β value of 0.276 and 0.289, with *p* < 0.001 respectively. The sensitivity analysis manifested the association between the use of Reyanning mixture and outcome was consistent with our main finding (Supplementary Table S1).

Furthermore, we included cases within the age range specified for each subgroup based on the age stratification, conducted independent analysis with multivariate Cox proportional-hazards model and presented in Table 4 to estimate the impact of using Reyanning mixture on the hospitalization duration and viral shedding time. The result still exhibited a significant association between the use of Reyanning mixture and hospitalization duration (HR: 1.844, 95% CI: 1.455-2.338, *p* < 0.001; HR: 1.590, 95% CI: 1.268-1.993, *p* < 0.001; HR: 1.788, 95% CI: 1.201-2.663, *p* < 0.05, respectively) as well as viral shedding time (HR: 1.547, 95% CI: 1.220-1.961, *p* < 0.001; HR: 1.349, 95% CI: 1.075-1.691, *p* < 0.05; HR: 1.534, 95% CI: 1.035-2.274, *p* < 0.05, respectively) in different age stratums.

**TABLE 4:** Cox proportional-hazards model for estimating the impact of using Reyanning mixture on the hospitalization duration and viral shedding time in different age stratums.

	Hospitalization duration				Viral shedding time			
	Univariate		Multivariate		Univariate		Multivariate	
	HR (95% CI)	p-value	HR (95% CI)	p-value	HR (95% CI)	p-value	HR (95% CI)	p-value
60–64 years	1.647 (1.352, 2.005)	<0.001	1.844 (1.455, 2.338)	<0.001	1.395 (1.146, 1.698)	0.001	1.547 (1.220, 1.961)	<0.001
65–69 years	1.587 (1.272, 1.981)	<0.001	1.590 (1.268, 1.993)	<0.001	1.359 (1.090, 1.695)	0.006	1.349 (1.075, 1.691)	0.010
≥70 years	1.771 (1.198, 2.617)	0.004	1.788 (1.201, 2.663)	0.004	1.583 (1.074, 2.332)	0.020	1.534 (1.035, 2.274)	0.033

CI, confidence interval; HR, hazard ratio.

### Secondary outcome

Throughout the treatment process, the most frequently observed adverse events included diarrhea, muscle soreness, and insomnia in both groups (Table 5). No severity adverse event was observed in our series. In addition, no progressive deterioration or death was occurred in the treatment period of our series. Besides, six patients in the treatment group were experienced with slight diarrhea, which was considered as a possible association with Reyanning mixture use. After symptomatic treatment, all adverse events were alleviated.

**TABLE 5:** Adverse events in the Treatment group and Control group.

Adverse events	Treatment group	Control group	*p*-value
Diarrhea	12 (4.1%)	36 (4.4%)	0.821
Muscle soreness	10 (3.4%)	32 (3.9%)	0.697
Headache	3 (1.0%)	10 (1.2%)	0.784
Insomnia	20 (3.8%)	33 (4.0%)	0.055
Dizziness	4 (1.3%)	9 (1.1%)	0.720
Palpitation	2 (0.7%)	5 (0.6%)	0.896
Stomachache	5 (1.7%)	12 (1.5%)	0.777

## Discussion

In the present study, we analyzed the data of 1,102 elderly patients from our ward, infected with SARS-CoV-2 Omicron variant. Our findings revealed a strong correlation between the length of hospitalization and viral shedding time in elderly patients, and various factors including age, vaccination status, and the use of Reyanning mixture. Compared to the control group, the use of Reyanning mixture significantly reduced hospitalization duration and viral shedding time in elderly individuals. Additionally, our study analyzed the patients in different age stratums still manifested a reduction in hospitalization duration and viral clearance time with Reyanning mixture treatment. Furthermore, there was no significant adverse event observed during the use of Reyanning mixture, nor any deterioration of the health condition. These results suggested that Reyanning mixture was an effective and safe treatment for elderly patients with COVID-19.

Since the initial emergence of COVID-19 pandemic in late 2019, multiple variants of SARS-CoV-2 have been identified. In addition to respiratory symptoms, infection can also trigger a cytokine storm, lead to systemic inflammation and damage to multiple organs. The Omicron variant, designated B.1.1.529, was a heavily mutated strain which was deemed a matter of concern on 26 November 2021 by the World Health Organization, posed a high infection risk with serious repercussions (Araf et al., 2022). Despite significant progress in clinical diagnosis and treatment, there were still many uncertainties in the therapy for this variation, and the long-term protection efficacy of current vaccines against viral variants still remained controversies, especially for elderly patients (Meo et al., 2021). In addition, the variant Omicron has been recognized cause more infectious, medical system overload and exhaust, with continuous outbreaks (Shen et al., 2022).

In addition to conventional clinical treatment, a range of initiatives, such as clinical trials and observation, have been undertaken to discover the utility of TCM for COVID-19, especially for those with antiviral and anti-inflammatory properties (Zhang et al., 2022b; Guo et al., 2022), to find alternative approaches to managing the disease course. Furthermore, various botanical drugs, such as Xuebijing injection, Qingfei Paidu decoction, and Lianhua Qingwen capsule, have been investigated in this regard and provided evidences in treating with COVID-19 infection (Li et al., 2021a; Hu et al., 2021; Tianyu and Liying, 2021). Shi J. et al. (2020) performed a retrospective analysis of data from 234 patients diagnosed with COVID-19. The results revealed that patients who received TCM treatment within 3 days of hospital admission exhibited a significant reduction in hospitalization duration, disease course, and nucleic acid negative conversion time, compared to those who received TCM decoction after 7 days or more. Hu et al. (2021) reported that the application of Lianhua Qingwen capsule manifested a significantly increasing in the recovery rate, with shorting the median time of symptom recovery in their study. Zhang et al. (2021) conducted an evaluation of 25 patients diagnosed with mild or common COVID-19 who received treatment with Tanreqing capsule. The result indicated a significant reduction in negative conversion time compared to the group that received conventional Western medicine. Shi N. et al. (2020) reported a series of 782 patients with confirmed COVID-19 and treated with Qingfei Paidu decoction, investigated the association between recovery and the treatment initiation time among infected individuals. However, there is a scarcity of research specifically targeting the application of TCM in treating elderly patients with SARS-CoV-2 infection, which remains an important area of study that requires further research.

The Reyanning mixture has been authorized by the National Medical Products Administration of China, in which consists four kind of herbs with a 2:2:2:1 proportion by weight. Previous studies has shown the usage of Reyanning mixture in treating infectious diseases such as fever, pneumonia, tonsillitis suppurative, sore throat, acute pharyngitis, and acute bronchitis (Lyu et al., 2020; Lyu et al., 2022). In a clinical investigation conducted by Yang M. et al. (2020), a series of 49 patients diagnosed with common COVID-19 were enrolled. The administration of Reyanning mixture was found to significantly ameliorate the clinical symptoms and chest images from computed tomography of the COVID-19 patients. However, the study did not specifically examine the correlation between the use of Reyanning mixture and the hospitalization duration or nucleic acid conversion time in elderly patients. Additionally, it should be noted that the sample size was relatively small, with only 26 cases receiving Reyanning mixture. In terms of the botanical drug mechanisms, Bao et al. (2020) assessed the efficacy of Reyanning mixture in a mouse model that was designed to mimic the syndrome of human coronavirus pneumonia. The results demonstrated the effectiveness of Reyanning mixture in the improvement of lung lesions, autoimmune function, enhancing gastro-intestinal function, as well as reducing the expression of inflammatory factors. Han et al. (2021) used the network pharmacology approach, exhibited its anti-inflammation effects via regulating the cell proliferation, and the surviving pathways. Wang M. et al. (2020) employed network pharmacology in addition to molecular docking methodologies and forecast the potential application of Reyanning mixture in treating COVID-19. Their findings suggested that the active constituents of Reyanning mixture may exert their therapeutic effects by modulating various targets, including CD40LG, IL2, IL6, IL10, and CXCL10, CXCL8. Nonetheless, it should be noted that the studies still lacked sufficient experimental validation through *in vivo* and *in vitro* assessments.

Recently, we conducted a randomized controlled study comprising of 2,830 patients, and the result indicated that Reyanning mixture represented a safe and effective treatment option for promoting recovery from asymptomatic and mild SARS-CoV-2 Omicron infection, as well as accelerating virus clearance (Xu et al., 2023). As the result of current study, we provided further evidence for the conversion to negative nucleic acid status, as well as a potential efficacious option of TCM for treating COVID-19 in elderly patients. Based on another study we conducted previously (Xu et al., 2022a), the finding indicated that elderly patients had prolonged hospitalization duration and a higher risk of deterioration. Additionally, we observed a correlation between the appliance of TCM and a reduction in hospitalization duration. An earlier study has identified age as an independent risk factor that may prolong the time of viral clearance (Wang K. et al., 2020). Moreover, some scholars have suggested that prolonged viral shedding time may be primarily associated with chronic diseases and low immunity (Hao et al., 2020), which were more prevalent among elderly patients. The result of our present study demonstrated that, in comparison to the control group, a significant reduction in hospitalization time and viral clearance time was observed in the population receiving Reyanning mixture, which could provide potential advantage for COVID-19 treatment in elderly patients.

Our finding provided another evidence of Reyanning mixture and treatment option for elderly patients infected with SARS-CoV-2 Omicron variant. Nonetheless, the study had certain limitations that should not be neglected. Firstly, as a retrospective and observational investigation, it may be subject to inherent biases. Secondly, the research exclusively focused on patients with the Omicron variant in Shanghai, which may restrict the generalizability of the findings. Moreover, the study was restricted in specific population, and the use of conventional Western medicine in the treatment regimen may have confounded the result and introduced potential bias.

In conclusion, given the circumstance of elderly patient, how to respond to SARS-CoV-2 infection and protect the health condition of this population still requires attention and concern. The present finding suggested that Reyanning mixture was a safe and effective treatment option for our series, and provided evidence for the application of Reyanning mixture in the elderly population. Prospective clinical trials with larger sample, targeting specific population, and incorporating more indicators for monitoring and analyzing are needed, and further exploration should be conducted to reveal the underlying mechanism and function of Reyanning mixture.

## Data Availability

The original contributions presented in the study are included in the article/Supplementary Material, further inquiries can be directed to the corresponding authors.

## Abbreviations

TCM, Traditional Chinese medicine; COVID-19, Coronavirus disease 2019; HR, Hazard ratio; CI, Confidence interval; Ct, Cycle Threshold; HPLC, High performance liquid chromatography; ORF, Open reading frame; N, Nucleocapsid.
