# Supplementary material for: A retrospective study of Reyanning mixture in elderly patients infected with SARS-CoV-2 Omicron variant
Source: Front Pharmacol. 2023 Jul 20;14:1185122. doi: 10.3389/fphar.2023.1185122 (PMC10399593; doi:10.3389/fphar.2023.1185122)
Supplement: Supplementary file 1 [file Table1.docx]

**Supplementary Table SI. Sensitivity analysis of the hospitalization duration and viral shedding time.**

|  | Hospitalization duration | | | | Viral shedding time | | | |
| --- | --- | --- | --- | --- | --- | --- | --- | --- |
|  | **Model 1** | | **Model 2** | | **Model 1** | | **Model 2** | |
| Variables | ***β value*** | ***p value*** | ***β value*** | ***p value*** | ***β value*** | ***p value*** | ***β value*** | ***p value*** |
| Group | 0.433 | <0.001 | 0.446 | <0.001 | 0.276 | <0.001 | 0.289 | <0.001 |
| Age | -0.040 | <0.001 | -0.038 | <0.001 | -0.039 | <0.001 | -0.036 | <0.001 |
| Gender | - | - | -0.003 | 0.967 | - | - | 0.010 | 0.867 |
| Type | - | - | 0.136 | 0.080 | - | - | 0.114 | 0.152 |
| Underlying diseases | - | - | -0.054 | 0.417 | - | - | -0.036 | 0.589 |
| Vaccination status | - | - | 0.186 | 0.005 | - | - | 0.165 | 0.013 |
